# A hundred years of activated sludge: time for a rethink

**DOI:** 10.3389/fmicb.2014.00047

**Published:** 2014-03-03

**Authors:** Abdul R. Sheik, Emilie E. L. Muller, Paul Wilmes

**Affiliations:** Eco-Systems Biology Group, Luxembourg Centre for Systems Biomedicine, University of LuxembourgEsch-sur-Alzette, Luxembourg

**Keywords:** activated sludge, wastewater microbial diversity and function, integrated omics, niche engineering, sustainability and renewable resources, energy reclamation – biodiesel and bioethanol, nutrient recovery and fertilizers, wastewater biorefinery column concept

## Abstract

Biological wastewater treatment plants (BWWTPs) based on the activated sludge (AS) process have dramatically improved worldwide water sanitation despite increased urbanization and industrialization. However, current AS-based operations are considered economically and environmentally unsustainable. In this Perspective, we discuss our current understanding of microbial populations and their metabolic transformations in AS-based BWWTPs in view of developing more sustainable processes in the future. In particular, much has been learned over the course of the past 25 years about specialized microorganisms, which could be more comprehensively leveraged to recover energy and/or nutrients from wastewater streams. To achieve this, we propose a bottom-up design approach, focused around the concept of a “wastewater biorefinery column”, which would rely on the engineering of distinct ecological niches into a BWWTP in order to guarantee the targeted enrichment of specific organismal groups which in turn will allow the harvest of high-value resources from wastewater. This concept could be seen as a possible grand challenge to microbial ecologists and engineers alike at the centenary of the discovery of the AS process.

Modern society is placing considerable strain on essential resources including water and energy (e.g., fossil fuels). Human interferences in biogeochemical cycles has already substantially altered the structure and function of atmospheric, aquatic, and terrestrial ecosystems (Vitousek, 1994). This is projected to worsen over the coming decades, as current estimates predict the human population to grow to more than 9 billion by 2050 (United Nations, 2013). In particular, waste residues may become pollutants if care is not given to their discharge into the environment. Thus, apart from public health considerations, systematic waste management is a central premise of sustainable development.

One hundred years ago, Ardern and Lockett (1914) described the process of activated sludge (AS) for biological remediation of sewage, based on heterotrophic microbial biomass either assimilating or oxidizing dissolved organic matter in influent wastewater. Following an aeration phase, the suspended biomass is separated from the treated wastewater by gravity filtration with a subsequent recycling of the majority of the sludge which is termed activated. To date, numerous variations of the original AS-based biological wastewater treatment plants (BWWTPs) are being operated world-wide including for enhanced biological phosphorus removal (Gujer et al., 1995), nitrification-denitrification (Henze et al., 1999), and anaerobic oxidation of ammonium (Van Dongen et al., 2001) together with anaerobic digestion of excess sludge (Grady et al., 2011). However, given the need for aeration as well as moving biomass between treatment tanks, AS-based BWWTPs consume considerable amounts of fossil fuel-derived energy resulting in considerable anthropogenic greenhouse gas emissions. In addition, the processes themselves also generate potent greenhouse gases such as CH_4_ (El-Fadel and Massoud, 2001) and N_2_O (Kampschreur et al., 2008). In this light, the current reliance on AS-based processes is environmentally unsustainable.

Although the organic and inorganic composition of wastewater depends on the influent (either municipal or industrial), it is estimated that the amount of chemical energy contained within wastewater is at least 10-fold higher than the amount of energy currently used to treat it (Shizas and Bagley, 2004; Heidrich et al., 2010). To date, energy recovery yields from anaerobic digestion of excess AS or from microbial fuel cells (MFCs) are significantly lower than the actual chemical energy contained within wastewater (Pham et al., 2006). Therefore, future sustainable strategies to reclaim energy from wastewater should not only reduce our dependence on fossil fuels but could also meet our demands of daily resources such as plastics and fertilizers.

In this Perspective, we introduce the concept of a “wastewater biorefinery column”, an approach for potentially leveraging the existing microbiological and biochemical knowledge of AS-based processes in a bottom-up design approach. This concept is based on the engineering of distinct ecological niches into future wastewater treatment processes, which may in turn allow the targeted enrichment of distinct organismal groups and the comprehensive recovery of energy and biotechnologically relevant molecules.

## THE CURRENT MICROBIOLOGICAL AND BIOCHEMICAL KNOWLEDGE OF AS-BASED PROCESSES

**FIGURE 1:**
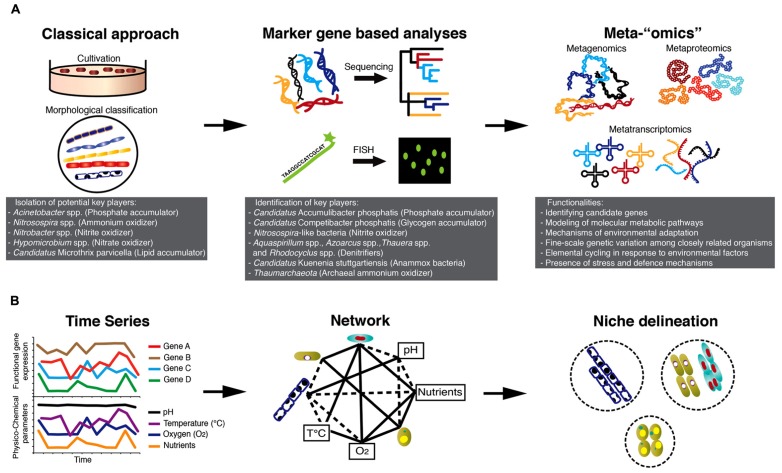
**Technology-driven understanding of the microbiology of AS-based processes. (A)** Advances in microbial ecology methods starting with cultivation-based approaches leading to community “omics” allowing the unveiling of the microbial “black box” of AS-based processes. **(B)** Integrated high-throughput “omics” together with recorded physico-chemical parameters of BWWTPs hold great promise to define niches of individual community members and their elemental transformation capabilities. The combined information may be used to engineer niches of specific microbial communities for subsequent energy/nutrient recovery.

Microbial communities of BWWTPs have for a long time been viewed as “black boxes”, as their structure and function have remained largely unknown. The majority of early studies on the microbiology of BWWTPs involved classical isolation techniques (Prakasam and Dondero, 1967) and standard light microscopy for morphological identification of specific bacterial groups, e.g., filamentous bacteria (Eikelboom, 1975; **Figure 1A**). Using classical culture-dependent microbiological techniques, *Acinetobacter* spp. was implicated in phosphorus removal (Fuhs and Chen, 1975), *Nitrosospira *spp.**was considered to be the key ammonium oxidizer (Prosser, 1989), *Nitrobacter* spp. (Henze et al., 1997) to be the dominating nitrite oxidizer and *Hypomicrobium* spp. to be a key denitrifier (Timmermans and Van Haute, 1983).

Intense research based on the retrieval of 16S rRNA gene sequences from BWWTPs in the last decades have ruled out the involvement of *Acinetobacter *spp. as a major player in phosphorus removal (Wagner et al., 1994; Kämpfer et al., 1996; Bond et al., 1995; Santos et al., 1999). So far, an uncultured and unclassified genus belonging to the *Beta-proteobacteria*, named *Candidatus* Accumulibacter phosphatis (CAp), has been shown to be a dominating phosphorus accumulating organism (PAO) in laboratory-scale reactors (Hesselmann et al., 1999; Crocetti et al., 2000) as well as in full-scale BWWTPs (Zilles et al., 2002; Wong et al., 2005). Moreover, subsequent molecular investigations have identified glycogen accumulating organisms (GAOs; Mino et al., 1995) as major competitors of CAp in anaerobic/aerobic sludge cycling. This includes a novel group belonging to *Gamma-proteobacteria* named as *Candidatus* Competibacter phosphatis (Crocetti et al., 2002) and other groups belonging to *Alpha-proteobacteria* (Wong et al., 2004).

Regarding the cycling of nitrogen, new key players identified using molecular approaches included diverse populations of ammonium oxidizers (Juretschko et al., 1998; Purkhold et al., 2000; Daims et al., 2001), *Nitrospira*-like microorganisms as dominant nitrite oxidizers (Juretschko et al., 1998; Daims et al., 2001; Dionisi et al., 2002; Maixner et al., 2006; Spieck et al., 2006) and denitrifiers belonging to the genera *Aquaspirillum, Azoarcus, Thauera*, and PAOs such as CAp**(Kong et al., 2004; Hesselsoe et al., 2005; Thomsen et al., 2007). More recently, archaeal**members of the *Thaumarchaeota* were also found to be capable of catalyzing the aerobic oxidation of ammonium (Park et al., 2006). The complexity of nitrogen cycling in BWWTPs was further brought to the forefront when distinct microorganisms having novel physiological properties, i.e., being able to carry out anaerobic ammonium oxidation (anammox) were identified (Mulder et al., 1995). These organisms belong to the phylum *Planctomycetes *(Strous et al., 1999)**and have been putatively named**“*Candidatus* Kuenenia stuttgartiensis” (Schmid et al., 2000).

Numerous investigations into the ecophysiology of microorganisms in BWWTPs were carried out using fluorescence *in situ* hybridization coupled with microautoradiography (MAR-FISH), which allowed direct visualization of specific microorganisms and qualitatively link specific carbon (C), nitrogen (N), and phosphorus (P) substrate transformations (Nielsen and Nielsen, 2002; Wagner et al., 2006). MAR-FISH allowed for example the study of competition between PAOs and GAOs for carbon substrates and allowed the formulation of models (Oehmen et al., 2010). During anaerobic conditions, PAOs assimilate organic carbon substrates, e.g., volatile fatty acids and store them as polyhydroxyalkanoates (PHAs; Kong et al., 2004). When exposed to aerobic conditions, PAOs oxidize PHAs, which provides energy for polyphosphate accumulation, leading to P removal from the wastewater by biomass retrieval. GAOs compete with PAOs for volatile fatty acids under anaerobic conditions for PHA synthesis (Bengtsson et al., 2008) and do not accumulate polyphosphate under aerobic conditions but rather use energy for glycogen accumulation (Crocetti et al., 2002; Kong et al., 2006). Further information on the intra-cellular accumulation of PHA or polyphosphate indicating storage and/or cycling of these polymers was confirmed by combining MAR-FISH with staining methods (Crocetti et al., 2000; Wong et al., 2004). Other MAR-FISH studies demonstrated the physiological potential of lipid accumulating organisms (LAOs), such as *Candidatus *Microthrix parvicella**to take up and store lipid substrates under anaerobic conditions and highlighted a competitive advantage of these filamentous bacteria compared to other organisms unable to assimilate these energy-rich substrates anaerobically (Andreasen and Nielsen, 1998; Nielsen et al., 2002).

Remarkably, advances in high-resolution molecular approaches, so called “meta-omics”, are now allowing concrete linkages to be drawn between microbial community compositions and functions. For example, metagenomic studies have unraveled some of the enigmatic microbial phenotypic traits by identifying candidate genes for ladderane biosynthesis and biological hydrazine metabolism in *Candidatus *Kuenenia stuttgartiensis**(Strous et al., 2006). Full-scale BWWTP metagenomic studies have underlined the need for more reference genomes of key species (Albertsen et al., 2011), which can be used as a template to interpret *in situ* data as illustrated by recent laboratory studies (Kristiansen et al., 2013; McIlroy et al., 2013). In this context, metagenomic or single cell sequencing data may be fruitfully employed to infer media formulation and growth conditions for organisms of interest.

While metagenomic studies have greatly contributed to the clarification of the functional capabilities of specific populations of interest, metaproteomics (Wilmes et al., 2008), metatranscriptomics (Yu and Zhang, 2012), and the combination of these two approaches (Haroon et al., 2013) have furthered our knowledge about the actual expression of relevant genes involved in key processes under different environmental conditions such as alternating anaerobic/aerobic phases. Thus, integrated “omics” over space and time (Muller et al., 2013) in combination with recorded physico-chemical parameters will allow reconstruction of the ecological networks and detailed definition of organismal niches (**Figure 1B**). Such knowledge may then be used to identify key determinants of overall microbial community structure and function, which in turn may be harnessed for comprehensive reclamation of energy and biotechnologically relevant products from wastewater.

## INTO THE FUTURE: USING THE INTELLIGENCE OF WASTEWATER MICROBES TO RECOVER THE HIDDEN TREASURE

Given the numerous organismal groups which have now been identified, as well as the short-term prospect of additional unprecedented data from multi-omic analyses, the time is ripe at the centenary of the AS-process to think about formulation of new biological wastewater treatment processes. In particular, we can start to consider bottom-up design approaches rather than the top-down approaches promulgated thus far.

**FIGURE 2:**
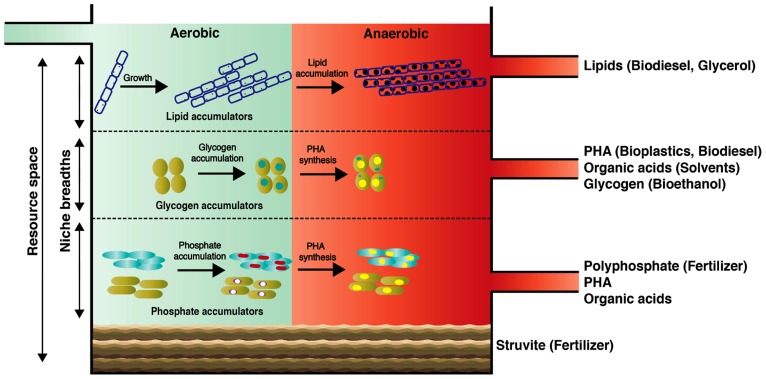
**Conceptual scheme of the proposed “wastewater biorefinery column” concept for dedicated resource recovery through engineered organismal niches. Our proposed concept may serve as a future grand challenge for microbial ecologists and engineers to tackle current AS process marking the centenary of its discovery**.

Here, we introduce the concept of a “wastewater biorefinery column” (**Figure 2**), based on a hypothetical bottom-up design approach, which takes into account the detailed knowledge of how specific microorganisms behave *in situ* and accumulate different storage compounds of interest over changing BWWTPs environmental conditions. Consequently, the engineering of specific niches would allow the harvest of high-value resources from wastewater. Such niche engineering may be achieved for example by (1) establishing distinct substrate gradients within the entire resource space of wastewater thereby exploiting the individual organismal niche breadths; (2) manipulation of the vertical distribution of key microorganisms by exploiting their respective settling velocities which in turn may be influenced by microbial/floc size and intracellular storage compounds; (3) harvesting dominant members for energy and/or nutrient reclamation. In laboratory-scale reactors, pronounced organismal enrichments have already been obtained for organisms of interest, in particular PAOs and GAOs (Winkler et al., 2011a), highlighting the feasibility of enriching these organisms by providing them with appropriate environmental conditions. With the advent of advanced meta-omic approaches, we will be able to define the niches of the respective organismal groups much more precisely, thereby allowing these to be engineering into future systems.**Given the heterogeneous and dynamic composition of wastewater, niches may have to be continually adjusted. Such niche fine-tuning could be based on feedback from microbial fuel cells acting as biosensors (Di Lorenzo et al., 2009), which would allow the continuous monitoring of organic and inorganic composition of influents.

Among the renewable sources of energy currently being explored, biodiesel holds significant promise as a potential alternative to partially replace petroleum-based fuels. At present, up to 85% of the overall biodiesel production cost is associated with the feedstocks (Knothe et al., 2005; Mondala et al., 2009). Consequently, biomolecules from wastewater of immediate bioenergy interest are saponifiable lipids since they can be easily transformed into biodiesel (Fukuda et al., 2001; Chisti, 2007; Mondala et al., 2009). In municipal wastewater, lipids can represent up to 41% of the total organic pools (Raunkjae, 1994), with a vast majority being triacylglycerides (TAGs) and a minor part free long chain fatty acids (Quemeneur and Marty, 1994). Importantly, the long chain fatty acids comprising TAGs within wastewater sludge are predominantly in the range of C_14_–C_18_ which are ideal for the production of methyl esters (Dufreche et al., 2007; Mondala et al., 2009). Due to their hydrophobicity, wastewater lipids are usually sorbed onto particles and not readily extractable (Dueholm et al., 2000). However, LAOs excrete extracellular lipases, which allow hydrolysis of lipids and efficient bacterial assimilation. Following our “wastewater biorefinery column” concept, the buildup of filamentous LAO biomass could be favored at the top of the column (**Figure 2**). Given the high levels of lipid accumulation by filamentous organisms and straightforward production of biodiesel from this lipid-rich biomass, a large potential exists in wastewater biodiesel production as its synthesis is economically viable (Knothe et al., 2005). Interestingly, a byproduct of TAG-derived biodiesel production is glycerol, which can be furthered used to produce PHA (Mothes et al., 2007), thereby leading to a complete and high-value valorization of the wastewater TAG fraction.

PHA synthesis naturally occurs within the microbial biomass of AS. Wastewater-derived PHAs are currently being used to synthesize biodegradable bioplastics on an industrial scale (Chen, 2009; Morgan-Sagastume et al., 2010) and they exhibit similar thermomechanical properties compared to chemically synthesized polypropylenes (Curran, 1996). Additionally, PHAs can be chemically transformed into the biofuel hydroxybutyrate methyl ester by acid catalyzed hydrolysis (Zhang et al., 2009). Therefore, wastewater-derived PHAs represent a suitable renewable resource for plastic production, as resource expenses in the entire PHA production chain can account up to 50% of the total production costs (Choi and Lee, 1999). The microbial accumulation of PHAs from wastewater can be very rapid (ca. 5 h) and pronounced (PHAs can constitute up to 77% of cell dry weight (Jiang et al., 2012). Both PAOs and GAOs are known to accumulate PHAs under alternating anaerobic/aerobic conditions. Given tailored environmental conditions, targeted enrichment of these organisms would therefore allow reclamation of PHA and fermentation products along with polyphosphate or glycogen (see below). PAOs in full-scale BWWTPs can contribute up to 35% of the total bacterial biomass (Oehmen et al., 2010) and laboratory reactor studies suggest that PAOs exhibit fast settling rates when compared to GAOs because of their differing cell densities (Vlaeminck et al., 2010; Winkler et al., 2011b; Volcke et al., 2012). These properties could be harnessed for separating both organismal groups and carrying out targeted resource reclamation (**Figure 2**). Through the establishment of density gradients, e.g., through manipulation of settling times, it could therefore be envisaged that GAOs could occupy the middle layers followed by PAOs in the lower section of the “wastewater biorefinery column” (**Figure 2**).

Although poorly studied in AS-based BWWTPs, fermentative bacteria contribute to the hydrolysis of complex organic macromolecules into low-molecular-weight substrates providing energy and carbon sources to other microbes. Produced molecules include industrially relevant commodities such as propionic acid, lactic acid, acetic acid, and formic acid (Kong et al., 2008). Interestingly, the production of alcohols and/or organic acids using fermentative treatment phases could be combined with biodiesel and bioplastic production since both processes require organic solvents. Most importantly, harvesting glycogen from GAO biomass could also be used for bioethanol production, an important biofuel (Hahn-Hägerdal et al., 2006).

Renewable fertilizer production using nutrient recovery from wastewater (primarily N and P) could cover up to 30% of the current agricultural fertilizer demand (Verstraete et al., 2009). A global estimate suggests that fertilizer production consumes up to 1.2% of the world’s energy (out of which 92.5% for N and 3% for P), contributing about 1.2% of the total anthropogenic greenhouse gas emissions (Kongshaug, 1998). In particular, PAO-rich biomass holds great promise as fertilizer. A major limitation of its current use as an agricultural fertilizer is that the biomass is typically rich in heavy metals (Veeken and Hamelers, 1999). However, there is increasing evidence that wastewater derived adsorbents (e.g., blast furnace slag) possesses high heavy metal adsorption capacities and offers a low-cost alternative treatment of metal-contaminated wastewater (Kurniawan et al., 2006) prior to further processing of the wastewater.

Apart from PAO-enriched biomass, struvite (MgNH_4_PO_4_·6H_2_O), a commonly occurring mineral found as precipitates in BWWTPs (Rawn et al., 1939) is a commercially produced fertilizer (Mavinic et al., 2007). Estimates suggest that within BWWTPs, 100 m^3^ of wastewater can yield up to 1 kg of struvite (Shu et al., 2006). The low solubility and the presence of high abundances of N and P in struvite is advantageous as a fertilizer since it reduces nutrient run-off in turn limiting eutrophication in receiving water bodies (Shu et al., 2006; El Diwani et al., 2007). Presence of low concentrations of suspended solids and high concentrations of ammonium and phosphate enhances struvite production (Doyle and Parsons, 2002). Thus, accelerating struvite production by retaining high levels of these ions in the wastewater while continuously reclaiming biomass fractions, e.g., from fast-growing populations could be another aim to be achieved in the “wastewater biorefinery column.”

In the context of N recovery for fertilizer production, nitrate accumulating organisms which are known to occur in varied habitats (McHatton et al., 1996; Schulz et al., 1999) may also be of pronounced interest. Although nitrate accumulators in AS-processes have so far not yet been described, they could represent a significant N source for fertilizer production. A selective enrichment strategy (Spieck et al., 2006) may provide a plausible approach to identify potential nitrate accumulators within full-scale AS-based BWWTPs.

In the past few decades, increasing environmental concerns have triggered the formulation and development of new strategies for energy and nutrient recovery from wastewater through AS-based processes (Verstraete et al., 2009). However, the yields in terms of recovery of either energy and/or biotechnological resources have so far been limited. Our proposed concept of a “wastewater biorefinery column” would use the existing and future wealth of information concerning the genetic repertoire of microorganisms and their metabolic transformations for sustainable production of bioenergy, bioplastics and fertilizers. However, in order to have this come to fruition, it is essential that we first obtain detailed descriptions of the niches of the individual community members. Once such knowledge has been obtained, wastewater treatment processes should be (re-)engineered taking into account the individual organismal niches using bottom-up design approaches rather the top-down strategies pursued so far. The optimization of processes may involve an iterative bottom-up design approach based on a discovery-driven planning approach (Muller et al., 2013) which would involve systematic omic analyses based on which ecological niches may be fine-tuned and, thus, the process continually tweaked to guarantee optimal resource recovery. We still have a long way to go to bring this vision to fruition but it may represent a grand challenge for microbial ecologists and engineers to tackle at the centenary of the discovery of the AS process.

## Conflict of Interest Statement

The authors declare that the research was conducted in the absence of any commercial or financial relationships that could be construed as a potential conflict of interest.

## References

[B1] Albertsen M. Hansen L. B. S. Saunders A. M. Nielsen P. H. Nielsen K. L. (2011). A metagenome of a full-scale microbial community carrying out enhanced biological phosphorus removal. *ISME J.* 6 1094–1106 10.1038/ismej.2011.176 22170425 PMC3358022

[B2] Andreasen M. Nielsen P. (1998). *In situ* characterization of substrate uptake by *Microthrix parvicella* using microautoradiography. *Water Sci. Technol.* 37 19–26 10.1016/S0273-1223(98)00079-1

[B3] Ardern E. Lockett W. T. (1914). Experiments on the oxidation of sewage without the aid of filters. *J. Soc. Chem. Ind.* 33 523–539 10.1002/jctb.5000331005

[B4] Bengtsson S. Werker A. Welander T. (2008). Production of polyhydroxyalkanoates by glycogen accumulating organisms treating a paper mill wastewater. *Water Sci. Technol.* 58 323–330 10.2166/wst.2008.381 18701781

[B5] Bond P. L. Hugenholtz P. Keller J. Blackall L. L. (1995). Bacterial community structures of phosphate-removing and non-phosphate-removing activated sludges from sequencing batch reactors. *Appl. Environ. Microbiol.* 61 1910–1916 7544094 10.1128/aem.61.5.1910-1916.1995 PMC167453

[B6] Chen G. Q. (2009). A microbial polyhydroxyalkanoates (PHA) based bio-and materials industry. *Chem. Soc. Rev.* 38 2434–2446 10.1039/b812677c 19623359

[B7] Chisti Y. (2007). Biodiesel from microalgae. *Biotechnol. Adv.* 25 294–306 10.1016/j.biotechadv.2007.02.001 17350212

[B8] Choi J. Lee S. Y. (1999). Factors affecting the economics of polyhydroxyalkanoate production by bacterial fermentation. *Appl. Microbiol. Biotechnol.* 51 13–21 10.1007/s002530051357

[B9] Crocetti G. R. Banfield J. F. Keller J. Bond P. L. Blackall L. L. (2002). Glycogen-accumulating organisms in laboratory-scale and full-scale wastewater treatment processes. *Microbiology* 148 3353–3364 12427927 10.1099/00221287-148-11-3353

[B10] Crocetti G. R. Hugenholtz P. Bond P. L. Schuler A. Keller J. Jenkins D. (2000). Identification of polyphosphate-accumulating organisms and design of 16S rRNA-directed probes for their detection and quantitation. *Appl. Environ. Microbiol.* 66 1175–1182 10.1128/AEM.66.3.1175-1182.2000 10698788 PMC91959

[B11] Curran M. A. (1996). Environmental life-cycle assessment. *Int. J. Life Cycle Assess.* 1 179 10.1007/BF02978949 PMC9607803 36320786

[B12] Daims H. Nielsen J. L. Nielsen P. H. Schleifer K.-H. Wagner M. (2001). *In situ* characterization of *Nitrospira*-like nitrite-oxidizing bacteria active in wastewater treatment plants. *Appl. Environ. Microbiol.* 67 5273–5284 10.1128/AEM.67.11.5273-5284.2001 11679356 PMC93301

[B13] Dionisi H. M. Layton A. C. Harms G. Gregory I. R. Robinson K. G. Sayler G. S. (2002). Quantification of *Nitrosomonas oligotropha*-like ammonia-oxidizing bacteria and *Nitrospira* spp. from full-scale wastewater treatment plants by competitive PCR. *Appl. Environ. Microbiol.* 68 245–253 10.1128/AEM.68.1.245-253.2002 PMC126567 11772633

[B14] Di Lorenzo M. Curtis T. P. Head I. M. Scott K. (2009). A single-chamber microbial fuel cell as a biosensor for wastewaters. *Water Res.* 43 3145–3154 10.1016/j.watres.2009.01.005 19482326

[B15] Doyle J. D. Parsons S. A. (2002). Struvite formation, control and recovery. *Water Res.* 36 3925–3940 10.1016/S0043-1354(02)00126-4 12405401

[B16] Dueholm T. Andreasen K. Nielsen P. (2000). Transformation of lipids in activated sludge. *Water Sci. Technol.* 43 165–172 11379087

[B17] Dufreche S. Hernandez R. French T. (2007). Extraction of lipids from municipal wastewater plant microorganisms for production of biodiesel. *J. Am. Oil Chem. Soc.* 84 181–187 10.1007/s11746-006-1022-4

[B18] Eikelboom D. H. (1975). Filamentous organisms observed in activated sludge. *Water Res.* 9 365–388 10.1016/0043-1354(75)90182-7

[B19] El Diwani G. El Rafie S. El Ibiari N. N. El-Aila H. I. (2007). Recovery of ammonia nitrogen from industrial wastewater treatment as struvite slow releasing fertilizer. *Desalination* 214 200–214 10.1016/j.desal.2006.08.019

[B20] El-Fadel M. Massoud M. (2001). Methane emissions from wastewater management. *Environ. Pollut.* 114 177–185 10.1016/S0269-7491(00)00222-0 11504340

[B21] Fuhs G. W. Chen M. (1975). Microbiological basis of phosphate removal in the activated sludge process for the treatment of wastewater. *Microb. Ecol.* 2 119–138 10.1007/BF02010434 24241234

[B22] Fukuda H. Kondo A. Noda H. (2001). Biodiesel fuel production by transesterification of oils. *J. Biosci. Bioeng.* 92 405–416 10.1016/S1389-1723(01)80288-7 16233120

[B23] Grady C. P. L. Jr. Daigger G. T. Love N. G. Filipe C. D. M Leslie Grady C. P. (2011). *Biological Wastewater Treatment*. London: IWA Publishing

[B24] Gujer W. Henze M. Mino T. Matsuo T. Wentzel M. C Marais G. V. R. (1995). The activated sludge model No. 2: Biological phosphorus removal. *Water Sci. Technol.* 31 1–11 10.1016/0273-1223(95)00175-M

[B25] Hahn-Hägerdal B. Galbe M. Gorwa-Grauslund M. F. Liden G. Zacchi G. (2006). Bio-ethanol–the fuel of tomorrow from the residues of today. *Trends Biotechnol.* 24 549–556 10.1016/j.tibtech.2006.10.004 17050014

[B26] Haroon M. F. Hu S. Shi Y. Imelfort M. Keller J. Hugenholtz P. (2013). Anaerobic oxidation of methane coupled to nitrate reduction in a novel archaeal lineage. *Nature* 500 567–570 10.1038/nature12375 23892779

[B27] Heidrich E. S. Curtis T. P. Dolfing J. (2010). Determination of the internal chemical energy of wastewater. *Environ. Sci. Technol.* 45 827–832 10.1021/es103058w 21142001

[B28] Henze M. Gujer W. Mino T. Matsuo T. Wentzel M. C Van Loosdrecht M. C. M. (1999). Activated sludge model no. 2d, ASM2d. *Water Sci. Technol.* 39 165–182 10.1016/S0273-1223(98)00829-4

[B29] Henze M. Harremoës P. Cour J. J. L Arvin E. eds (1997). *Wastewater Treatment. Biological and Chemical Processes* 2nd Edn Berlin: Springer-Verlag 10.1007/978-3-662-22605-6

[B30] Hesselmann R. P. X. Werlen C. Hahn D. van der Meer J. R Zehnder A. J. B. (1999). Enrichment, phylogenetic analysis and detection of a bacterium that performs enhanced biological phosphate removal in activated sludge. *Syst. Appl. Microbiol.* 22 454–465 10.1016/S0723-2020(99)80055-1 10553298

[B31] Hesselsoe M. Nielsen J. L. Roslev P. Nielsen P. H. (2005). Isotope labeling and microautoradiography of active heterotrophic bacteria on the basis of assimilation of ^14^CO_2_. *Appl. Environ. Microbiol.* 71 646–655 10.1128/AEM.71.2.646-655.2005 15691913 PMC546759

[B32] Jiang Y. Marang L. Tamis J. Van Loosdrecht M. C. M. Dijkman H. Kleerebezem R. (2012). Waste to resource: Converting paper mill wastewater to bioplastic. *Water Res.* 46 5517–5530 10.1016/j.watres.2012.07.028 22921584

[B33] Juretschko S. Timmermann G. Schmid M. Schleifer K.-H. Pommerening-Röser A. Koops H.-P. (1998). Combined molecular and conventional analyses of nitrifying bacterium diversity in activated sludge: *Nitrosococcus mobilis* and *Nitrospira*-like bacteria as dominant populations. *Appl. Environ. Microbiol.* 64 3042–3051 9687471 10.1128/aem.64.8.3042-3051.1998 PMC106813

[B34] Kämpfer P. Erhart R. Beimfohr C. Böhringer J. Wagner M. Amann R. (1996). Characterization of bacterial communities from activated sludge: culture-dependent numerical identification versus *in situ* identification using group-and genus-specific rRNA-targeted oligonucleotide probes. *Microb. Ecol.* 32 101–121 10.1007/BF00185883 8688004

[B35] Kampschreur M. J. van der Star W. R. L. Wielders H. A. Mulder J. W. Jetten M. S. M Van Loosdrecht M. C. M. (2008). Dynamics of nitric oxide and nitrous oxide emission during full-scale reject water treatment. *Water Res.* 42 812–826 10.1016/j.watres.2007.08.022 17920100

[B36] Knothe G. Van Gerpen J. H. Krahl J. (2005). *The Biodiesel Handbook*. Champaign, IL: AOCS press 10.1201/9781439822357

[B37] Kong Y. Nielsen J. L. Nielsen P. H. (2004). Microautoradiographic study of *Rhodocyclus*-related polyphosphate-accumulating bacteria in full-scale enhanced biological phosphorus removal plants. *Appl. Environ. Microbiol.* 70 5383–5390 10.1128/AEM.70.9.5383-5390.2004 15345424 PMC520863

[B38] Kong Y. Xia Y. Nielsen J. L. Nielsen P. H. (2006). Ecophysiology of a group of uncultured gammaproteobacterial glycogen-accumulating organisms in full-scale enhanced biological phosphorus removal wastewater treatment plants. *Environ. Microbiol.* 8 479–489 10.1111/j.1462-2920.2005.00914.x 16478454

[B39] Kong Y. Xia Y. Nielsen P. H. (2008). Activity and identity of fermenting microorganisms in full-scale biological nutrient removing wastewater treatment plants. *Environ. Microbiol.* 10 2008–2019 10.1111/j.1462-2920.2008.01617.x 18557773

[B40] Kongshaug G. (1998). “Energy consumption and greenhouse gas emissions in fertilizer production,” in *IFA Technical Conference Marrakech.* Available at:

[B41] Kristiansen R. Nguyen H. T. T. Saunders A. M. Nielsen J. L. Wimmer R. Le V. Q. (2013). A metabolic model for members of the genus *Tetrasphaera* involved in enhanced biological phosphorus removal. *ISME J.* 7 543–554 10.1038/ismej.2012.136 23178666 PMC3578573

[B42] Kurniawan T. A. Chan G. Lo W. Babel S. (2006). Comparisons of low-cost adsorbents for treating wastewaters laden with heavy metals. *Sci. Total Environ.* 366 409–426 10.1016/j.scitotenv.2005.10.001 16300818

[B43] Maixner F. Noguera D. R. Anneser B. Stoecker K. Wegl G. Wagner M. (2006). Nitrite concentration influences the population structure of *Nitrospira* – like bacteria. *Environ. Microbiol.* 8 1487–1495 10.1111/j.1462-2920.2006.01033.x 16872410

[B44] Mavinic D. S. Koch F. A. Huang H. Lo K. V. (2007). Phosphorus recovery from anaerobic digester supernatants using a pilot-scale struvite crystallization process. *J. Environ. Eng. Sci.* 6 561–571 10.1139/S07-007

[B45] McHatton S. C. Barry J. P. Jannasch H. W. Nelson D. C. (1996). High nitrate concentrations in vacuolate, autotrophic marine *Beggiatoa* spp. *Appl. Environ. Microbiol.* 62 954–958 16535282 10.1128/aem.62.3.954-958.1996 PMC1388807

[B46] McIlroy S. J. Kristiansen R. Albertsen M. Michael Karst S. Rossetti S. Lund Nielsen J. (2013). Metabolic model for the filamentous “*Candidatus* Microthrix parvicella” based on genomic and metagenomic analyses. *ISME J.* 7 1161–1172 10.1038/ismej.2013.6 23446830 PMC3660683

[B47] Mino T. Liu W.-T. Kurisu F. Matsuo T. (1995). Modelling glycogen storage and denitrification capability of microorganisms in enhanced biological phosphate removal processes. *Water Sci. Technol.* 31 25–34 10.1016/0273-1223(95)00177-O

[B48] Mondala A. Liang K. Toghiani H. Hernandez R. French T. (2009). Biodiesel production by *in situ* transesterification of municipal primary and secondary sludges. *Bioresour. Technol.* 100 1203–1210 10.1016/j.biortech.2008.08.020 18809323

[B49] Morgan-Sagastume F. Karlsson A. Johansson P. Pratt S. Boon N. Lant P. (2010). Production of polyhydroxyalkanoates in open, mixed cultures from a waste sludge stream containing high levels of soluble organics, nitrogen and phosphorus. *Water Res.* 44 5196–5211 10.1016/j.watres.2010.06.043 20638096

[B50] Mothes G. Schnorpfeil C. Ackermann J. (2007). Production of PHB from crude glycerol. *Eng. Life Sci.* 7 475–479 10.1002/elsc.200620210

[B51] Mulder A. Graaf A. A. Robertson L. A. Kuenen J. G. (1995). Anaerobic ammonium oxidation discovered in a denitrifying fluidized bed reactor. *FEMS Microbiol. Ecol.* 16 177–184 10.1111/j.1574-6941.1995.tb00281.x

[B52] Muller E. E. L. Glaab E. May P. Vlassis N. Wilmes P. (2013). Condensing the omics fog of microbial communities. *Trends Microbiol.* 21 325–333 10.1016/j.tim.2013.04.009 23764387

[B53] Nielsen J. Nielsen P. (2002). Quantification of functional groups in activated sludge by microautoradiography. *Water Sci. Technol.* 46 389–395 12216655

[B54] Nielsen P. Roslev P. Dueholm T. Nielsen J. (2002). *Microthrix parvicella*, a specialized lipid consumer in anaerobic-aerobic activated sludge plants. *Water Sci. Technol.* 46 73–80 12216691

[B55] Oehmen A. Carvalho G. Lopez-Vazquez C. M. Van Loosdrecht M. C. M Reis M. A. M. (2010). Incorporating microbial ecology into the metabolic modelling of polyphosphate accumulating organisms and glycogen accumulating organisms. *Water Res.* 44 4992–5004 10.1016/j.watres.2010.06.071 20650504

[B56] Park H.-D. Wells G. F. Bae H. Criddle C. S. Francis C. A. (2006). Occurrence of ammonia-oxidizing archaea in wastewater treatment plant bioreactors. *Appl. Environ. Microbiol.* 72 5643–5647 10.1128/AEM.00402-06 16885322 PMC1538709

[B57] Pham T. H. Rabaey K. Aelterman P. Clauwaert P. De Schamphelaire L. Boon N. (2006). Microbial fuel cells in relation to conventional anaerobic digestion technology. *Eng. Life Sci.* 6 285–292 10.1002/elsc.200620121

[B58] Prakasam T. B. S. Dondero N. C. (1967). Aerobic heterotrophic bacterial populations of sewage and activated sludge I. Enumeration.* Appl. Microbiol.* 15 461–467 10.1128/am.15.3.461-467.1967 PMC546943 5340649

[B59] Prosser J. I. (1989). Autotrophic nitrification in bacteria. *Adv. Microb. Ecol.* 30 125 10.1016/s0065-2911(08)60112-5 2700538

[B60] Purkhold U. Pommerening-Röser A. Juretschko S. Schmid M. C. Koops H.-P. Wagner M. (2000). Phylogeny of all recognized species of ammonia oxidizers based on comparative 16S rRNA and *amoA* sequence analysis: implications for molecular diversity surveys. *Appl. Environ. Microbiol.* 66 5368–5382 10.1128/AEM.66.12.5368-5382.2000 11097916 PMC92470

[B61] Quemeneur M. Marty Y. (1994). Fatty acids and sterols in domestic wastewaters. *Water Res.* 28 1217–1226 10.1016/0043-1354(94)90210-0

[B62] Raunkjær K. Hvitved-Jacobsen T. Nielsen P. H. (1994). Measurement of pools of protein, carbohydrate and lipid in domestic wastewater. *Water Res.* 28 251–262 10.1016/0043-1354(94)90261-5

[B63] Rawn A. M. Banta A. P. Pomeroy R. (1939). Multiple-stage sewage sludge digestion. *Trans. Am. Soc. Civ. Eng.* 104 93–119

[B64] Santos M. M. Lemos P. C. Reis M. A. M. Santos H. (1999). Glucose metabolism and kinetics of phosphorus removal by the fermentative bacterium *Microlunatus phosphovorus*. *Appl. Environ. Microbiol.* 65 3920–3928 10473396 10.1128/aem.65.9.3920-3928.1999 PMC99721

[B65] Schmid M. Twachtmann U. Klein M. Strous M. Juretschko S. Jetten M. (2000). Molecular evidence for genus level diversity of bacteria capable of catalyzing anaerobic ammonium oxidation. *Syst. Appl. Microbiol.* 23 93–106 10.1016/S0723-2020(00)80050-8 10879983

[B66] Schulz H. N. Brinkhoff T. Ferdelman T. G. Mariné M. H. Teske A. Jørgensen B. B. (1999). Dense populations of a giant sulfur bacterium in Namibian shelf sediments. *Science* 284 493–495 10.1126/science.284.5413.493 10205058

[B67] Shizas I. Bagley D. M. (2004). Experimental determination of energy content of unknown organics in municipal wastewater streams. *J. Energy Eng.* 130 45–53 10.1061/(ASCE)0733-9402(2004)130:2(45)

[B68] Shu L. Schneider P. Jegatheesan V. Johnson J. (2006). An economic evaluation of phosphorus recovery as struvite from digester supernatant. *Bioresour. Technol.* 97 2211–2216 10.1016/j.biortech.2005.11.005 16364632

[B69] Spieck E. Hartwig C. McCormack I. Maixner F. Wagner M. Lipski A. (2006). Selective enrichment and molecular characterization of a previously uncultured *Nitrospira* – like bacterium from activated sludge. *Environ. Microbiol.* 8 405–415 10.1111/j.1462-2920.2005.00905.x 16478447

[B70] Strous M. Fuerst J. A. Kramer E. H. M. Logemann S. Muyzer G. van de Pas-Schoonen K. T. (1999). Missing lithotroph identified as new planctomycete. *Nature* 400 446–449 10.1038/22749 10440372

[B71] Strous M. Pelletier E. Mangenot S. Rattei T. Lehner A. Taylor M. W. (2006). Deciphering the evolution and metabolism of an anammox bacterium from a community genome. *Nature* 440 790–794 10.1038/nature04647 16598256

[B72] Thomsen T. R. Kong Y. Nielsen P. H. (2007). Ecophysiology of abundant denitrifying bacteria in activated sludge. *FEMS Microbiol. Ecol.* 60 370–382 10.1111/j.1574-6941.2007.00309.x 17391331

[B73] Timmermans P Van Haute A. (1983). Denitrification with methanol: fundamental study of the growth and denitrification capacity of *Hyphomicrobium* sp. *Water Res.* 17 1249–1255 10.1016/0043-1354(83)90249-X

[B74] United Nations, Department of Economic and Social Affairs, Population Division (2013). World Population Prospects: The 2012 Revision, Highlights and Advance Tables. ESA/P/WP.228

[B75] Van Dongen U. Jetten M Van Loosdrecht M. C. M. (2001). The SHARON-Anammox process for treatment of ammonium rich wastewater. *Water Sci. Technol.* 44 153–160 11496667

[B76] Veeken A. H. M Hamelers H. V. M. (1999). Removal of heavy metals from sewage sludge by extraction with organic acids. *Water Sci. Technol.* 40 129–136 10.1016/S0273-1223(99)00373-X

[B77] Verstraete W. Van de Caveye P. Diamantis V. (2009). Maximum use of resources present in domestic “used water.” *Bioresour. Technol.* 100 5537–5545 10.1016/j.biortech.2009.05.047 19577923

[B78] Vitousek P. M. (1994). Beyond global warming: ecology and global change. *Ecology* 75 1861–1876 10.2307/1941591

[B79] Vlaeminck S. E. Terada A. Smets B. F. De Clippeleir H. Schaubroeck T. Bolca S. (2010). Aggregate size and architecture determine microbial activity balance for one-stage partial nitritation and anammox. *Appl. Environ. Microbiol.* 76 900–909 10.1128/AEM.02337-09 19948857 PMC2813012

[B80] Volcke E. I. P. Picioreanu C. De Baets B van Loosdrecht M. C. M. (2012). The granule size distribution in an anammox-based granular sludge reactor affects the conversion – implications for modeling. *Biotechnol. Bioeng.* 109 1629–1636 10.1002/bit.24443 22252967

[B81] Wagner M. Erhart R. Manz W. Amann R. Lemmer H. Wedi D. (1994). Development of an rRNA-targeted oligonucleotide probe specific for the genus *Acinetobacter* and its application for *in situ* monitoring in activated sludge. *Appl. Environ. Microbiol.* 60 792–800 7512807 10.1128/aem.60.3.792-800.1994 PMC201394

[B82] Wagner M. Nielsen P. H. Loy A. Nielsen J. L. Daims H. (2006). Linking microbial community structure with function: fluorescence *in situ* hybridization-microautoradiography and isotope arrays. *Curr. Opin. Biotechnol.* 17 83–91 10.1016/j.copbio.2005.12.006 16377170

[B83] Wilmes P. Andersson A. F. Lefsrud M. G. Wexler M. Shah M. Zhang B. (2008). Community proteogenomics highlights microbial strain-variant protein expression within activated sludge performing enhanced biological phosphorus removal. *ISME J.* 2 853–864 10.1038/ismej.2008.38 18449217

[B84] Winkler M. K. H. Bassin J. P. Kleerebezem R. De Bruin L. M. M. Van den Brand T. P. H Van Loosdrecht M. C. M. (2011a). Selective sludge removal in a segregated aerobic granular biomass system as a strategy to control PAO–GAO competition at high temperatures. *Water Res.* 45 3291–3299 10.1016/j.watres.2011.03.024 21513967

[B85] Winkler M. K. H. Kleerebezem R. Kuenen J. G. Yang J Van Loosdrecht M. C. M. (2011b). Segregation of biomass in cyclic anaerobic/aerobic granular sludge allows the enrichment of anaerobic ammonium oxidizing bacteria at low temperatures. *Environ. Sci. Technol.* 45 7330–7337 10.1021/es201388t 21744798

[B86] Wong M.-T. Mino T. Seviour R. J. Onuki M. Liu W.-T. (2005). *In situ* identification and characterization of the microbial community structure of full-scale enhanced biological phosphorous removal plants in Japan. *Water Res.* 39 2901–2914 10.1016/j.watres.2005.05.015 15993461

[B87] Wong M.-T. Tan F. M. Ng W. J. Liu W.-T. (2004). Identification and occurrence of tetrad-forming *Alphaproteobacteria* in anaerobic–aerobic activated sludge processes. *Microbiology* 150 3741–3748 10.1099/mic.0.27291-0 15528660

[B88] Yu K. Zhang T. (2012). Metagenomic and metatranscriptomic analysis of microbial community structure and gene expression of activated sludge. *PLoS ONE* 7:e38183 10.1371/journal.pone.0038183 PMC3364235 22666477

[B89] Zhang X. Luo R. Wang Z. Deng Y. Chen G.-Q. (2009). Application of (R)-3-hydroxyalkanoate methyl esters derived from microbial polyhydroxyalkanoates as novel biofuels. *Biomacromolecules* 10 707–711 10.1021/bm801424e 19249855

[B90] Zilles J. L. Peccia J. Kim M.-W. Hung C.-H. Noguera D. R. (2002). Involvement of *Rhodocyclus*-related organisms in phosphorus removal in full-scale wastewater treatment plants. *Appl. Environ. Microbiol.* 68 2763–2769 12039731 10.1128/AEM.68.6.2763-2769.2002 PMC123978

